# On the Treatment of Purpura Hemorrhagica in the Horse with Argentum Colloidale Credé1Translated from the Berliner Thierärztliche Wochenschrift, November 17, 1898.

**Published:** 1899-05

**Authors:** W. Dieckerhoff

**Affiliations:** Berlin


### SELECTION.

### ON THE TREATMENT OF PURPURA HEMORRHAGICA IN THE HORSE WITH ARGENTUM COLLOIDALE CREDÉ.[1]

By Dr. W. Dieckerhoff,
BERLIN.

Privy-Councillor Dr. Credé, Physician-in-chief to the Carola Hospital at Dresden, has recently demonstrated the successful treatment of the diseases in the human subject caused by staphylococci and streptococci by means of soluble metallic silver.[2] The author first demonstrated that the citrate of silver (itrol) and lactate of silver (actol) are energetic and non-poisonous antiseptics; and his observations led him to suspect that suitable, readily soluble silver salts would be able to counteract, to a certain extent, general septic infections of the human body. He remarks as follows: " . . . That a successful treatment of the septic infections can only be conceived of when metallic silver in the fluid form can be introduced into the tissue fluids; in which event I assume that the antiseptically acting silver salts will be formed in the body itself."

A metallic silver has accordingly been prepared after Credé's directions, which is almost completely soluble in water and the albuminous fluids; and with this the Credé preparations are manufactured by the chemical factory of von Heyden, of Radebeul, near Dresden. The soluble silver preparations are to be employed in phlegmon (lymphangitis with lymphadenitis) in the human subject, and are as follows:

1. Unguentum Credé—containing 15 per cent. argentum colloidale, for silver inunctions.
2. Pilulæ argenti colloidalis—argentum colloidale, with sugar of milk and glycerin, for internal use.
3. Bacilli argenti colloidalis, for uterine maladies.
4. Solutio argenti colloidalis, in distilled water, 1 to 200 to 1000, for subcutaneous injection.

An intravenous injection of the watery solution of argentum colloidale has not as yet been tried in the human subject, though Credé calls attention to its possibility in the following words: " It is pos-

[1] Translated from the Berliner Thierärztliche Wochenschrift, November 17, 1898.

[2] B. Credé. "Silver as an External and an Internal Antiseptic," Archiv für klinische Chirurgie, vol. lv. No. 4. Ibid. "Soluble Metallic Silver as a Curative Agent," Klinisch-therapeutische Wochenschrift, Wien, 1898, Nos. 14 and 15.

sible that in severe cases of sepsis intravenous injection would be the most energetic and efficacious method of administration."

The advantages of silver in the treatment of phlegmonous and septic diseases in man have already been proved.[1] The histories published by Werler demonstrate the remarkable curative effects of the colloidal silver in acute sepsis, in chronic septic infection, and in the chronic multiple furunculosis of the human subject. They induced me to employ the Credé remedies experimentally for the internal diseases of the horse. I began my therapeutic experiments on the morbus maculosus (anasarca) of solipeds, since theoretical considerations would lead one to expect favorable results from the new remedy in it.

I cannot in this place enter upon the subject of the former incorrect diagnosis and nomenclature of the morbus maculosus of horses. It may be well known that I was the first to demonstrate the fact that the disease developed in consequence of the introduction of a special virus into the blood circulation of the animal.[2] A detailed description of the malady can be found in my *Text-book of Special Pathology and Therapeutics.* I mention there that it is not yet decided whether the microörganisms themselves or the poisons that they produce are the immediate cause of the appearance of the hemorrhagic foci. Even to-day this question is not entirely cleared up by actual investigation. There can be no doubt, however, that the disease often develops as a sequel to suppurative processes (purulent catarrh of the respiratory mucous membrane, abscesess, suppurating phlegmons).

In many other cases, however, it begins without any primary suppurative disease being demonstrable in the horse, in the so-called "latent or cryptogenetic" way. In these cases we must assume that the same virus which enters the blood from a demonstrable suppurative primary affection has reached the circulation from some place not accessible to our investigations.

As regards the characterization of the hemorrhages I will cite the following passage from my text-book: "The extravasated blood and the as yet unknown virulent substance found in it have a phlogogenous (inflammation exciting) effect on the tissues. In the subcutis there develops from the hemorrhagic foci a diffuse inflammation with an abundant exudation of serum and white

[1] Dr. Werler, of Berlin; "Soluble Metallic Silver as a Curative Agent," notice and report in Lassar's Dermatologische Zeitschrift, 1898, No. 3. Ibid., "Surgical Experiences with Soluble Metallic Silver in the Treatment of Septic Wound Infections," Deutsche medicinische Wochenschrift, 1898, No. 40,

[2] Pferdestaupe, 1882, p. 102.

blood-corpuscles. The exudate has a jelly-like consistency, and is yellowish or yellowish-white in color." Hence the inflammation of the connective tissue which occurs around the local bleedings in the subcutis and the mucosæ of the respiratory and digestive tract is comparable to the erysipelatous phlegmon (diffuse suppurative phlegmon) of the fetlock; and it may be supposed that in the occurrence of the hemorrhages and their consequences the strepto-cocci are etiologically concerned. These considerations led me in 1886 to attempt its cure by the introduction of iodine into the circulation (by the intratracheal injection of Lugol's solution), with the purpose of destroying the specific cause of the disease. The happy influence of this treatment upon the course of the disease I reported upon in a paper that was published in Adam's *Wochenschrift*, 1887, No. 12. Although I have since that time employed the iodine-therapy in many cases, and very often with advantage, in addition to the symptomatic treatment, I cannot deny that it is often ineffective, more especially when the disease is of idiopathic origin and some unattainable purulent collection in an internal organ of the body is not present. The poisoning also of horses suffering from morbus maculosus by the iodine is often not complete enough to prevent the formation of new hemorrhagic foci and the enlargement of the foci already present during the next few days.

Hence the idea of experimenting with the soluble metallic silver in the treatment of the disease was a welcome one to me. I did not attempt the introduction of the remedy into the stomach or by inunction, or by the subcutaneous method, for it seemed indubitable to me that intravenous injection was the most suitable method to combat the disease producers or their toxins in the blood or the internal organs.

A few preliminary experiments upon old horses convinced me that the injection of a 1 per cent. solution of argentum colloidale did not irritate the vein upon which it was done, and caused no other disturbances. I determined upon a provisional dosage of 0.5 grammes ($7\frac{1}{2}$ grains), so that at each intravenous injection of the sick horse 50 grammes ($1\frac{2}{3}$ ounces) of the 1 per cent. solution was injected. I used a syringe of 25 grammes ($\frac{5}{6}$ ounces) capacity, filling it twice for each injection, while the canula remained *in situ*. In the four cases here recorded the results from the new remedy were most excellent, and they even exceeded my expectations.

Case I.—Mare, nine or ten years old, a heavily-built work-horse, had suffered for eight days from a mild pharyngeal catarrh; was sent to the clinic October 12, 1898, because the hind legs had been œdematous for several days.

Condition on October 13th : Pulse 60, respirations 12, temperature 39.2° C. (102.5° F.). Petechiæ and hemorrhagic suffusions on the nasal mucous membranes of both sides. On the bridge of the nose the skin is swollen to the size of the palm of one's hand. On the forearms of both forelimbs are tumor-shaped, œdematous swellings of the subcutis. Both hind legs show a diffuse, œdematous swelling of the skin. Appetite bad.

*Treatment.* Repeated washings of the hemorrhagic foci in the skin with Burrows' solution, with the addition of camphor. At intervals of two hours the horse received a solution of 0.5 grammes ($7\frac{1}{2}$ grains) argentum colloidale dissolved in 50 grammes ($1\frac{2}{3}$ ounces) of distilled water injected into the jugular vein.

By October 14th the petechiæ in the nose had become paler, and the swelling of the limbs showed the first signs of retrogression. In the following days a normal appetite returned, and on October 27th the horse was dismissed from the clinic entirely cured.

Case II.—A six-year-old brown gelding, a heavy work-horse, was sent to the clinic on October 16, 1898, with great swelling of the prepuce and prolapse of the penis, and bloody undermining of the nasal mucous membrane, and isolated swellings of the skin of the head and of the fore limbs. Pulse 56, respirations 12, temperature 39.1° C. (102.3° F.). Appetite moderate. On account of the symptoms the diagnosis of morbus maculosus was made.

On October 17th the condition of the horse, which had been placed in a box-stall, was not much changed. On October 18th 0.5 gramme ($7\frac{1}{2}$ grains) of argentum colloidale in 50 grammes ($1\frac{2}{3}$ ounces) of distilled water was injected into the left jugular vein. Local treatment for the swellings of the penis and sheath was also employed.

The illness got better in one day, and by October 25th had entirely disappeared.

Case III.—A work-horse (mare), took sick on October 19, 1898, with the symptoms of morbus maculosus, and was brought to the clinic the next day. On October 21st examination revealed the following : Pulse 40, respiration 20, temperature 39.8° C. (103.6° F.). Little appetite. Bloody swellings of the nasal mucosæ and of the subcutis of the skin on the upper parts of the limbs. Besides local washings with Burrows' solution, on October 21st and 22d, there was each time injected by the intravenous method 0.5 gramme ($7\frac{1}{2}$ grains) of argentum colloidale dissolved in 50 grammes ($1\frac{2}{3}$ ounces) of distilled water. By October 23d already all the important manifestations of the purpura were gone. On October 25th the owner took the horse away.

Case IV.—On the morning of November 9, 1898, a black mare, about sixteen years old, a light draught-horse, which had swellings of the hind limbs for several days and colic for two hours, was brought to the clinic. The examination was made at 9 A.M. on November 9th. Extensive bloody undermining and swelling of the nasal mucous membranes, marked swelling of the skin over the nostrils, on the cheek, and at the margins of the lips on both sides. Eyelids tumefied. Both the palpebral and ocular conjunctivæ infiltrated with blood. As the skin of the false nostrils was swollen, also, inspiration was accomplished with a snuffling sound. There were colicky pains, so great that the horse could hardly be kept on his feet. Intestinal sounds were absent. Limbs were not swollen. The horse could take neither food nor water, both chewing and deglutition being interfered with. Pulse 45, respirations 18, temperature 39.3° C. (102.6° F.).

The case was a specially severe one, as was shown by the extensive swelling of the head, the colic, and the dysphagia. The symptoms showed that the intestinal as well as the respiratory mucous membrane had lately developed fresh hemorrhagic foci. My former experience led me to judge that none of the usual remedies nor the iodine treatment could save the horse. The case seemed especially suitable for the determination of the question of the value of the silver treatment.

On November 9th, from 9 o'clock in the morning until 7 o'clock in the evening, the animal received five injections of 0.5 gramme ($7\frac{1}{2}$ grains) of the remedy at intervals of two hours. Thus within ten hours 2.5 grammes ($37\frac{1}{2}$ grains) of argentum colloidale were injected into the jugulars.

The colicky symptoms ceased after the first two injections. In the afternoon the horse seemed a little livelier; it stuck its mouth into the bucket of water, but could not yet swallow any of it. The snoring respiration continued, but did not get worse.

On November 10th, at 9 A.M., examination already showed a retrogression of the most important symptoms. Pulse 48, respirations 13, temperature 38.1° C. (100.5° F.). The horse could drink water, but the swelling and inflammation of the subcutis of both cheeks and lips still prevented the taking and chewing of food. I ordered two injections of 0.5 gramme ($7\frac{1}{2}$ grains) each of argentum colloidale during the day.

On the afternoon of November 10th the horse could chew flat slices of turnip placed upon his tongue, and could swallow them. In the evening it took a little oats.

On November 11th, in the morning, the horse seemed bright and had a good appetite. She ate oats, hay, and turnips. The bloody undermining of the nasal mucosa was gradually clearing up, and the swellings of the skin on the head had already considerably retrogressed. Breathing free. Pulse 42, respirations 11, temperature 37.8° C. (100° F.).

Complete recovery followed in a few days. On November 12th and 13th there was still a slight discharge of thick muco-purulent material from both nostrils, which I attributed to the healing of the hemorrhagic foci in the pharyngeal mucosa. Otherwise the horse was free from all important symptoms of disease.

As the result of these observations I regard it as certain that the argentum colloidale Credé is a very efficacious remedy in purpura hemorrhagica of horses. In veterinary practice the intravenous injection of a 1 per cent. solution of the drug in distilled water is readily done, and is the most suitable method of administration—more especially where a rapid and vigorous general effect is required to hinder the further development of the disease. The exact dosage and the frequency of administration are questions that further and more extensive investigations must ultimately decide. That the horse can take relatively large doses of argentum colloidale without detriment is shown by the history of the fourth case recited above. Further clinical research must also settle the question as to how far the subcutaneous injection of a watery solution of the drug, which horses likewise stand very well, will fulfil the therapeutic indications of the disease.

It need hardly be insisted on that the remedy is useless for the complications of morbus maculosus (sloughing of the skin over the hemorrhagic foci on the limbs and head, laryngeal stenosis, and pneumonia), and that in general it can only develop its good effects when employed in the early stages of the disease.

The preliminary maladies so frequently present, the abscesses and the purulent catarrhs, will hardly be influenced by it. Treatment according to the symptomatic indications will be necessary in the future in many cases, in addition to the medication with the colloidale silver.

In addition to purpura the argentum colloidale will probably be found very useful in other general diseases of domesticated animals, more especially in the purulent and septic blood-poisonings. Perhaps it will also do good service in diffuse elephantiasis of the limbs of the horse. As in the human subject, so also in animals, phlegmonous inflammation of the subcutaneous cellular tissue after infected wounds will be efficiently combated by the method.

It had no influence upon the course of typical pneumonia in two cases in which I employed this method of intravenous injection.

Its price does not prevent the preparation being used in veterinary practice.

